# Chronic Kidney Disease as a Cardiovascular Disorder—Tonometry Data Analyses

**DOI:** 10.3390/ijerph191912339

**Published:** 2022-09-28

**Authors:** Mateusz Twardawa, Piotr Formanowicz, Dorota Formanowicz

**Affiliations:** 1 Institute of Computing Science, Poznan University of Technology, 60-965 Poznan, Poland; 2 ICT Security Department, Poznan Supercomputing and Networking Center Affiliated to the Institute of Bioorganic Chemistry, Polish Academy of Sciences, 61-139 Poznan, Poland; 3 Department of Medical Chemistry and Laboratory Medicine, Poznan University of Medical Sciences, 60-806 Poznan, Poland

**Keywords:** chronic kidney disease, cardiovascular disease, tonometry

## Abstract

Tonometry is commonly used to provide efficient and good diagnostics for cardiovascular disease (CVD). There are many advantages of this method, including low cost, non-invasiveness and little time to perform. In this study, the effort was undertaken to check whether tonometry data hides valuable information associated with different stages of chronic kidney disease (CKD) and end-stage renal disease (ESRD) treatment. For this purpose, six groups containing patients at different stages of CKD following different ways of dialysis treatment, as well as patients without CKD but with CVD and healthy volunteers were assessed. It was revealed that each of the studied groups had a unique profile. Only the type of dialysis was indistinguishable a from tonometric perspective (hemodialysis vs. peritoneal dialysis). Several techniques were used to build profiles that independently gave the same outcome: analysis of variance, network correlation structure analysis, multinomial logistic regression, and discrimination analysis. Moreover, to evaluate the classification potential of the discriminatory model, all mentioned techniques were later compared and treated as feature selection methods. Although the results are promising, it could be difficult to express differences as simple mathematical relations. This study shows that artificial intelligence can differentiate between different stages of CKD and patients without CKD. Potential future machine learning models will be able to determine kidney health with high accuracy and thereby classify patients. ClinicalTrials.gov Identifier: NCT05214872.

## 1. Introduction

Chronic kidney disease (CKD), defined as abnormalities in kidney damage or glomerular filtration rate (GFR) below 60 mL/min/1.73 $m^{2}$ that have been present for over three months and have an impact on health [1], is strongly associated with cardiovascular disease (CVD) [2].

The risk of developing CVD is 5 to 10 times higher in CKD than in healthy subjects, and the probability of CV events becomes significantly higher in patients even in the early stages of CKD compared to the general population. Interestingly, CKD patients are much more likely to experience CV death than reach the stage of end-stage renal disease (ESRD) [1].

This unique relationship between CKD and CVD is commonly explained by the cluster of CV risk factors found in CKD patients. These factors can be classified as (a) traditional, such as (1) advanced age, (2) diabetes mellitus, (3) hypertension, (4) dyslipidemia, and (b) non-traditional (CKD-specific), including (1) oxidative stress, (2) chronic inflammation, (3) immune deficiency, (4) anemia (reduction of erythrocytes lifespan, erythropoietin deficiency, and iron disturbances [3,4]), (5) proteinuria, (6) volume overload, (7) neuro-hormonal activation, increased sympathetic tone, (8) the impact of dialysis itself, (9) malnutrition (10) upregulation of the renin–angiotensin–aldosterone system, (11) increased salt sensitivity, and (12) calcium–phosphate balance disorders. As bone mineral metabolism worsens as CKD progresses, the associated hyperphosphatemia, secondary hyperparathyroidism, and inhibition of vitamin D synthesis lead to vascular calcification, causing hardening of the arteries [1,2,5,6]. All of these factors contribute to the structural changes in the heart and blood vessels with remodeling and stiffening of the arteries and the left ventricle.

The relationship between BP and stiffness is inconclusive because stiffening may directly affect the blood pressure (BP) profile along with higher systolic pressure and heart rate, but parallel increased arterial wall distension (associated with BP) may exacerbate stiffness.

Since hypertension and CKD frequently coexist [7], accurate BP measurement is critical to proper patient diagnosis and care. The usual approach to assessing the risk of hypertension is based on the measurements made by the arm cuff with the assumption that it is a reasonable representation of BP exposure to target organs. However, studies of invasive BP measurement in humans have shown that this is not necessarily the case, with the possibility of a high degree of variability in systolic blood pressure (SBP) measured in the aorta compared to peripheral limb arteries. Generally, hypertension diagnosis is based on BP in the arm. Still, the SBP response to vasoactive drugs can vary greatly between the aorta and the brachial artery. Arm cuff BP may overestimate cardiovascular risk; hence, central aortic BP predicting mortality could be a better method for patient management.

Given that central aortic BP more closely reflects pressure felt by organs, central BP is expected to be more clinically significant than BP on the brachial cuff. The CV risk prediction may be overstated by arm cuff BP. Indeed, this was shown with invasive central BP measurement, and taken together, these data suggest that greater precision in treating hypertension can be achieved by knowing a person’s central blood pressure, which can help guide care. Since intra-arterial blood pressure measurement is not widely applicable, techniques for non-invasive assessment of central arterial BP have been developed using various methods, including using a generalized transfer function to the radial BP curve of the radial artery recorded by applanation tonometry [8].

The study by Carlsen et al. [9] showed that advanced CKD is associated with serious vascular abnormalities altering the normal relationship of BP between the aorta and peripheral arteries and thus potentially affecting central BP measurement results. The main goal of their study was to determine the effect of CKD and aortic stiffness on the accuracy of non-invasive central BP measurement, with invasive central BP measurement as the reference standard. An interesting result was the significant trend to underestimate the central SBP with a decrease in the estimated GFR, even using the invasive calibration standard. On the other hand, a new finding was that the degree of central SBP underestimation was due to a gradual association with deteriorating renal function and an increase in aortic stiffness. Boutouyrie et al. [10] reviewed the merits and limits of the work by Carlsen et al., concluding that deeper studies are needed here.

In the absence of unequivocal results concerning the usefulness of the tonometry results among CKD patients, this study has focused on analyzing tonometry data collected from different groups, divided according to their CKD progression and the method of the ESRD treatment; two groups (healthy and suffering from CVD without CKD) acted as comparative groups. The main goal of this study was to determine the possibility of future patient classification based only on tonometry examination. For this purpose, CV profiles were created based on dimensionality reduction and feature selection techniques. Moreover, the correlation structure of parameters was analyzed for each group and compared due to dense relationships between variables that are inherent features of tonometry results.

## 2. Materials and Methods

### 2.1. Study Group

The case-control cross-sectional study involved 252 participants, including 183 patients with diagnosed CKD and 69 people without CKD. Recruitment to the study lasted two years.

During the 2-year follow-up period from inclusion to this study, each patient’s history of fatal cardiovascular events was recorded separately. The primary end point was fatal acute myocardial infarct (AMI), acute ischemic stroke, or any unexpected or sudden death, but only if an autopsy proved it to be CV-related. If the cause of death was in doubt or there was no contact with the patient within two years of study enrollment, these patients were excluded and not considered further.

Based on the presence or absence of CKD, the degree of CKD, and the type of renal replacement therapy used, six research groups were created. Allocation to the groups was based on the GFR calculated following the KDIGO 2012 recommendations according to the MDRD formula based on the measurement of serum creatinine concentration expressed in [mg/dL] eGFR = 186 × [creatinine concentration in mg/dL] − 1.154 × [age] − 0.203 (for females this formula has to be multiplied by 0.724).

The CKD group was selected from the patients of the Nephrology Outpatient Clinic, Peritoneal Dialysis Clinic, and the Dialysis Center at the Clinical Hospital. H. Święcicki in Poznań. Here, four subgroups were formed.

1. The hemodialyzed patients’ group, named HD (n = 71), included patients treated with hemodialysis (HD). HD procedures were performed on each patient three times a week via an arteriovenous fistula from their own or artificial vessels. The duration of HD was at least 10 h/week using standard bicarbonate dialysis fluids and polysulfone low-flux dialyzers. The blood flow during HD was 200–350 mL/min, with an average dialysis fluid flow of 500 mL/min.
2. The peritoneal dialyzed patients’ group, named PD (n = 35), included patients treated with peritoneal dialysis with standard lactate and glucose-based fluids. In this group, initially, due to the treatment technique, two subgroups were separated: (a) a group (n = 15) treated with the automatic peritoneal dialysis (ADO) technique in which the cycler performed an automatic fluid exchange at night in the peritoneal cavity, the mean exchange time was 10 h, and in some patients, an additional 7.5% icodextrin solution was used during the day; and (b) a group of patients (n = 20) using the technique of continuous cycling peritoneal dialysis (CAPD), performing 3–5 daily dialysis fluid changes in the peritoneal cavity, volume 1.5–2.5 liters. In some patients, an additional 7.5% icodextrin solution was used in a single, long exchange. When the subgroups were analyzed separately, no statistically significant differences between the two techniques were shown, possibly due to the small size of the groups; hence, the group of peritoneal dialysis patients was presented as a whole, i.e., the PD group.
3. The pre-dialyzed patients’ group, named PRE (n = 48), included patients in the pre-dialysis period (stage G3b-G4 CKD) with a moderate or severe decrease in eGFR (eGFR 44–29 mL/min/1.73 $m^{2}$).
4. Patients at the early stages of CKD formed a group named CKD1-2 (n = 29), which included patients at the stages G1-G2 CKD with a mild decrease in eGFR (eGFR >90–60 mL/min/1.73 $m^{2}$). The studies in this group were conducted to check the changes that occur due to CVD but without kidney disease.

Hypertensive nephropathy was the most common cause of CKD. The most commonly used medications included loop diuretics, angiotensin-converting enzyme inhibitors, angiotensin II receptor antagonists, calcium channel blockers, centrally acting antihypertensives (clonidine hydrochloride, methyldopa), alpha-blockers, calcium binders (calcium carbonate or calcium acetate), and vitamin D and its derivatives (alfacalcidol). Some patients also received drugs stimulating erythropoiesis by subcutaneous injection (patients in the PRE and DO) or intravenously (patients in the HD).

Apart from the CKD group, a non-CKD group was formed. It was created from two subgroups:

1. Cardiology patients’ group, named CARD (n = 37), including patients with a history of at least one CV event, admitted to the hospital (Department of Internal Medicine, Division of Cardiology–Intensive Therapy, Poznan University of Medical Sciences) for elective angiography, in whom both laboratory and clinical signs of impaired kidney function were revealed.
2. Healthy volunteers’ group, named CONTROL (n = 32), was composed of healthy people, with no evidence of impairment of renal and cardiovascular functions in their history and at the time of enrollment in the study. This group included people recruited from among those reporting to the laboratory for routine checkups.

**Figure 1:**
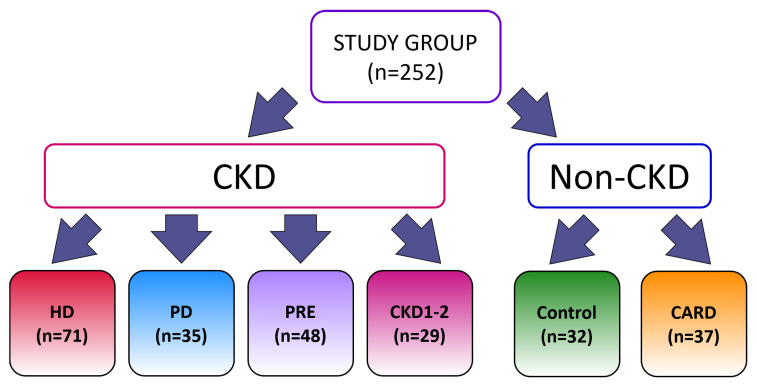
Division into groups selected for this study.

The exclusion criteria for all studied groups were as follows: active acute infection, immunosuppressive therapy, kidney transplant, abnormal liver function, malignant tumors within the last five years, and alcohol abuse within the last five years. The structure of the groups is shown in Figure 1.

### 2.2. Non-Invasive Cardiological Examinations

For non-invasive cardiological examinations, the following devices based on advanced algorithms for analyzing pulse waveforms and EKG curves were used: the Portapres TM (Finapres Medical System (FMS), Enschede, The Netherlands), Pulse Trace 2000 TM (Micro Medical Ltd., Rochester, Kent, UK), the SphygmoCor tonometer (ATCOR, Naperville, IL, USA), and the Colin blood pressure monitor (BPM)-7000 TM device (Colin Medical Instruments Corp., San Antonio, TX, the USA with a 10 MHz transducer).

The analyses were performed in the morning hours. After prior consultation with the attending physician, the persons participating in the study did not take their morning dose of medications. Initially, the examined person, previously connected to the monitoring equipment, rested for 15 min in a warm, air-conditioned, and dark room. Then, for 15 min, the waveform of the right hand’s pulse in the middle finger was continuously recorded using the PortapresTM device using the phenomenon of photoplethysmography. This device consists of a cuff that fits over the finger and a transducer placed on the test subject’s wrist. The cuff contains a diode that emits infrared light and a photocell that registers the amount of light that has not been absorbed. As it passes through the finger tissue, the emitted light is absorbed by the heme contained in the hemoglobin. The entire measurement is performed non-invasively, continuously, from beat to beat. This produces a pulse wave curve at finger level. Then, using a Pulse Trace 2000TM device, the change in pulse wave contour was analyzed based on the changes in blood volume in the fingertip. The Puls TraceTM device shows the shape of a typical pulse wave on the monitor, based on which the RI and SI indicators are calculated.

Central (aortic) systolic blood pressure was examined using the SphygmoCor Mx system (AtCor Medical, Australia). For this purpose, the applanation tonometry method was used with the Colin BMP 7000 device (Japan). The Colin device is placed on the wrist, which enables continuous recording of the pulse wave at the level of the radial artery. The resulting analog signal is then sent in real time to the SphygmoCor Mx device. This makes it possible to analyze the central (aortic) pulse wave shape.

The evaluation of the variables using the non-invasive devices was based on advanced algorithms for analyzing pulse waveforms and EKG curves. The SphygmoCor tonometer used derived the central aortic pressure waveform from cuff pulsations recorded at the brachial artery. Analysis of the waveforms provided the values of the key parameters, including:

- Heart rate (HR) [bpm],
- Ejection duration (ED) [ms],
- Peripheral systolic blood pressure (pSP) [mmHg],
- Peripheral diastolic blood pressure (pDP) [mmHg],
- Peripheral mean pressure (pMEANP, pMAP) [mmHg],
- Time to first peak (peripheral T1, pT1) [ms],
- Time to second peak (peripheral T2, pT2) [ms],
- Peripheral pressure at first peak (pP1) [ms],
- Peripheral pressure at second peak (pP2 [ms],
- Peripheral T1/ED% (pT1ED),
- Peripheral T2/ED% (pT2ED),
- Peripheral augmentation index (pAI) [%],
- Peripheral end systolic pressure (pESP) [mmHg],
- Central diastolic pressure (cDP) [mmHg],
- Central mean pressure (cMEANP, cMAP) [mmHg],
- Central pulse pressure (cPP) [mmHg],
- Time to first peak-Aortic (cT1) [ms],
- Time to second peak-Aortic (cT2) [ms],
- cT1R (time of the start of the reflected wave) [ms],
- Central pressure at first peak (cP1) [mmHg],
- Central pressure at second peak (cP2) [mmHg],
- T1/ED% control panels (cT1ED),
- Central T2/ED% (cT2ED),
- Central augmentation index (cAI) [%],
- Central end systolic pressure (cESP) [mmHg],
- Central augmented pressure (cAP) [mmHg],
- Central mean pressure of systole (cMPS) [mmHg],
- Central mean pressure of diastole (cMPD) [mmHg],
- Central tension time index (cTTI) [mmHg · s],
- Central diastolic pressure-time index (cDTI) [%],
- cSVI (SEVR) Buckberg Sub-Endocardial Viability Ratio [%],
- Central pulse period (cPERIOD) [ms],
- Central diastolic duration (cDD) [mmHg],
- Central pulse high (cPH) [mmHg],
- Central systolic pulse pressure (cPP) [mmHg],
- Central diastolic pressure (cDP) [mmHg],
- Time from systolic inflection point (if present) or systolic peak to diastolic inflection point (PPT).

Moreover, pulse trace TM enabled the assessment of the following variables:

- Reflection index (RI) [%],
- Vascular stiffness index (SI) [m/s],
- Peripheral pulse pressure (pPP) [mmHg],
- Peripheral pulse pressure/central pulse pressure (pPP/cPP ratio) [%].

A more detailed explanation of the methods of determining the selected variables can be found at https://atcormedical.com/technology/sphygmocor/ (accessed on 30 May 2022).

### 2.3. Ethics Statement

The study was conducted following the Helsinki Declaration of the World Medical Society and approved by the Bioethics Committee of the Poznan University of Medical Sciences (Decision No. 274/16 and 275/16, both of 3 March 2016). All enrolled study participants met the criteria and completed the study. They were fully informed about the study, and all gave written informed consent before enrollment.

### 2.4. Data Analysis

**Figure 2:**
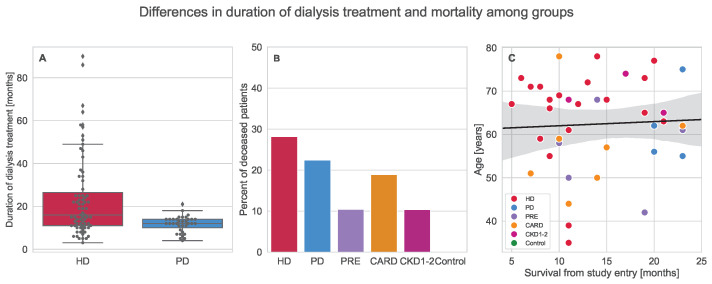
Dialysis duration and mortality within the groups: (**A**) the boxplot illustrating dialysis treatment duration with distinction of hemodialyzed (HD) and peritoneal dialyzed (PD) patients; (**B**) the barplot depicting the percent of deceased patients in every group with 2 year follow up; (**C**) the scatterplot presenting relation of age and number of months survived for dead patients, additional regression line was added to clarify the image and show lack of correlation (Spearman ρ = −0.075, *p* = 0.7194).

The following techniques were used to evaluate the potential of tonometry measurements for patient classification based on group profiles: analysis of variance, network correlation structure analysis, multinomial logistic regression, and discrimination analysis. General information about the groups is presented in Table 1 and Table 2. Moreover, additional data about dialysis duration and mortality are illustrated in Figure 2.

**Figure 3:**
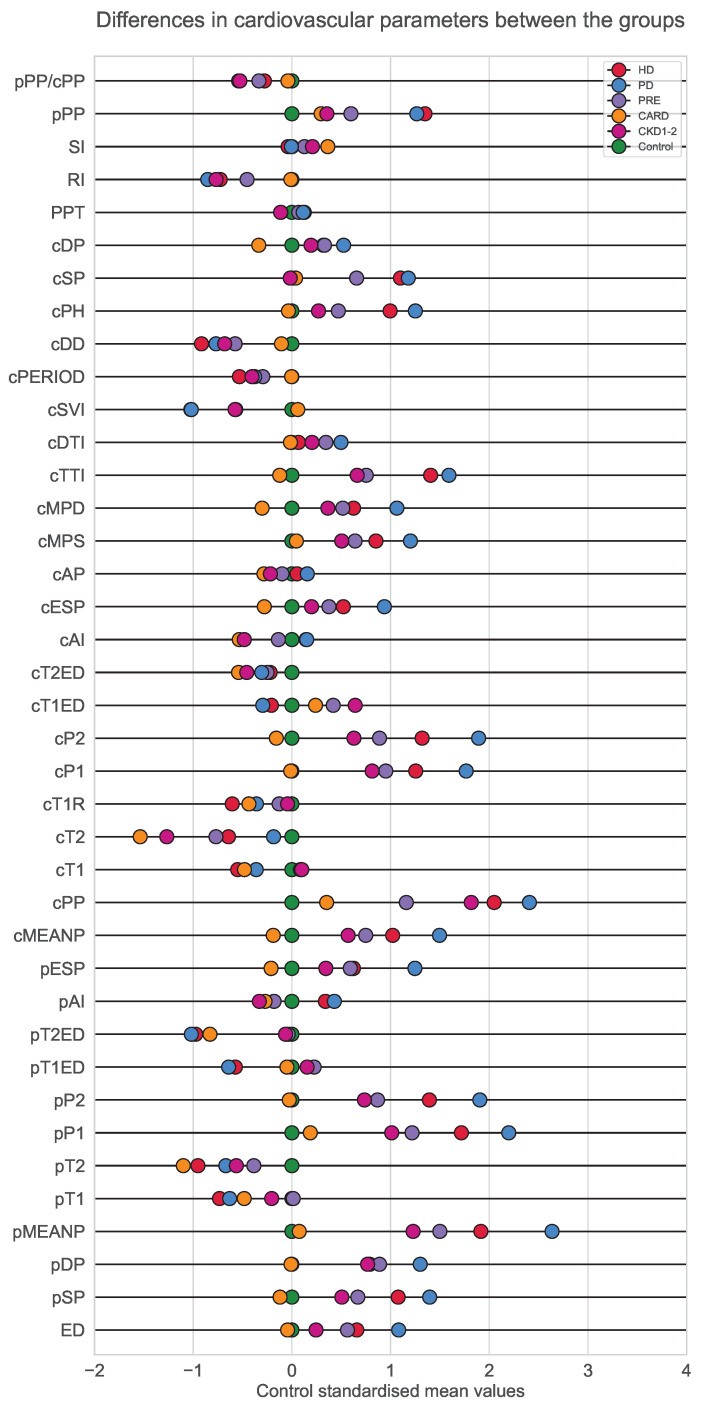
Visualization of differences between the groups made for the 39 cardiovascular parameters analyzed in this study. For each variable, values in the control group were standardized. Then, for each group, mean value transformation into a z-score according to standardization made for the control group was performed (each dot represents the z-scored average within a group). Each dot shows how much the mean parameter value calculated for the group was away from the healthy control average. The goal of the picture was to build intuition behind the direction and the magnitude of differences and similarities between the groups.

Analysis of variance was performed with the Kruskal–Wallis H test (see Table 3). Differences between groups for a given set of variables were visualized in the form of boxplots. Detailed boxplots for every variable analyzed can be found in Supplementary File S1 (Figures S1–S7). Moreover, to highlight and present differences in a compact and easy-to-read way, special z-score plots were made (Figure 3).

**Figure 4:**
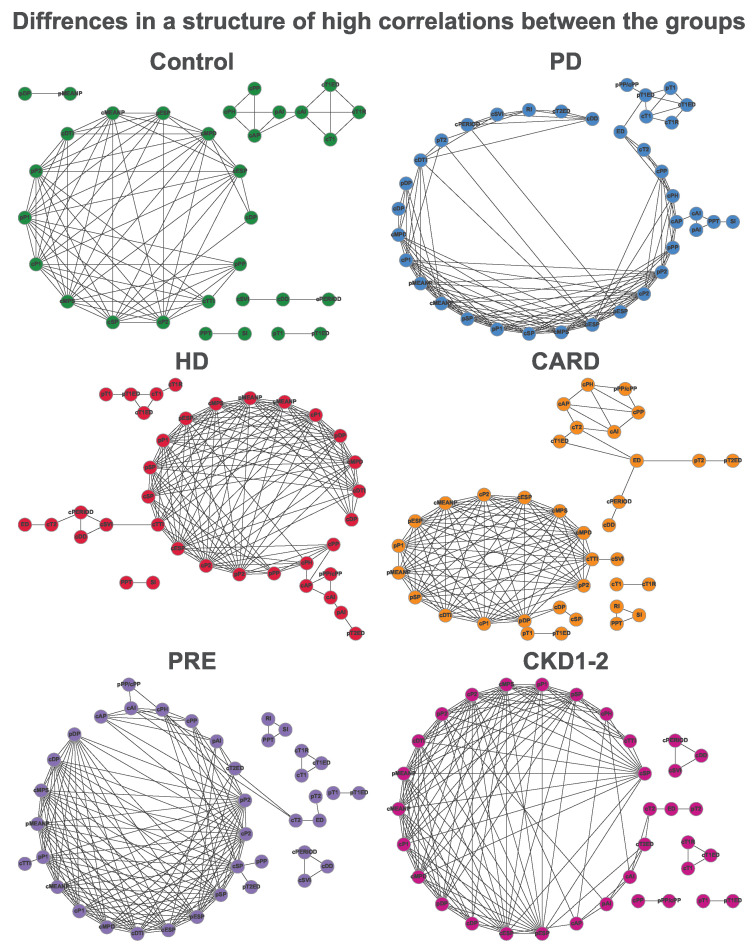
Correlation graphs representing only strong correlations between the parameters (Spearman ρ≥0.7 and p<0.01).

Since tonometric variables are connected in many ways, the network correlation structure of the data was explored. Spearman rank correlation for every variable was calculated and compared between groups. All group-specific correlations are reported in Supplementary File S1. Figure 4 presents highly correlated variables in the form of a graph (Spearman rho≥ 0.7 and *p* < 0.01). Table 4 presents node degree distribution for each group-specific strong correlation visualized as a network in Figure 4. Detailed information can be seen in correlation heatmaps that are included in Supplementary File S1 (Figures S8–S13). The full correlation analysis results, additional descriptive statistics, and mortality data are placed in Supplementary File S2.

To filter out redundant markers, a multinomial logit model was used. This step was able to limit the number of analyzed tonometric parameters, which was important due to a high number of strong correlations present in the data. To find a satisfactory subset of significant variables able to differentiate the groups of patients, step-wise selection based on Akaike information criterion (AIC) was executed. The final model results described by the Wald test can be found in Supplementary File S1 (Table S1).

The performance of six parameter arrangements, the five selected by previously presented methods plus the whole original set were evaluated in terms of Kernel Fisher Discrimination Analysis performance. Table 5 describes details of the selection techniques. Accuracy was evaluated 10 times (results are in the form of mean with standard deviation) for the randomly selected train (80%), and test (20%) sets. The mean and variance analysis seleccted 14 parameters (pSP, pMEANP, pP1, pP2, cMEANP, cPP, cP1, cP2, cMPS, cTTI, cSVI, cPH, cSP, pPP) based on the Kruskal–Wallis test and location pattern of the means on the standardized parameters plot (Figure 3). The means must create a general pattern that can be supported by statistical tests. In general, it is visible that HD and PD patients tend to be close together, as well as CKD1-2 with PRE and CARD and Control. The correlation graph analysis delivered two parameter sets, one for significant differences in node degrees (pSP, pDP, pMEANP, cDP, cTTI, cSP, RI, pPP) and the second for the variables with little correlation (ED, pT1, pT2, pT1ED, pT2ED, pAI, cPP, cT1, cT2, cT1R, cT1ED, cT2ED, cAI, cAP, cSVI, cPERIOD, cDD, cPH, PPT, RI, SI, pPP, pPP/cPP). Based on a suggestion taken from the multinomial regression model, 23 parameters were further analyzed, namely ED, pDP, pT2, pT1ED, pAI, cDP, cPP, cT1, cT1R, cT1ED, cAI, cESP, cAP, cMPD, cTTI, cPERIOD, cPH, cSP, PPT, RI, SI, pPP, and pPP/cPP. As it turned out, the last two parameter sets were very similar, so it was decided to run KFDA with the intersection of these two sets (ED, pT2, cT1ED, pAI, cPP, cT1, cT1R, pT1ED, cAI, cAP, cPERIOD, cPH, PPT, SI, RI, pPP, pPP/cPP). Interestingly, only one parameter was present in every generated set, i.e., pPP.

**Figure 5:**
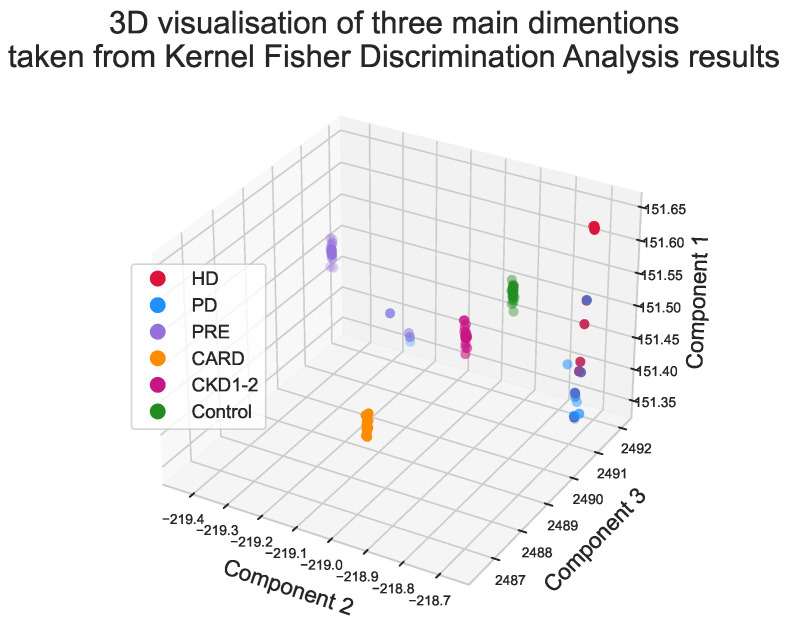
Kernel Fisher Discrimination Analysis results. Local clusters of patients from the same group are visible.

**Figure 6:**
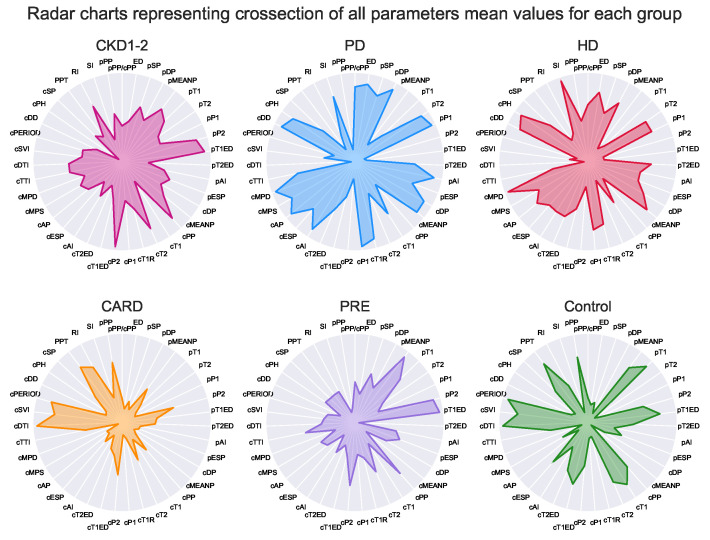
Original tonometric mean parameters values. The values for each individual parameter were standardized according to the scalar created for the whole patient population (n = 252) and normalized to fall between 0 and 1 values afterward. The radar plots consist of 39 tonometry parameters and show where the means inside every group lie (after scaling). The lowest values are in the circle center, and the highest touch the circumference. The white lines are provided to easily track values calculated for each parameter according to the label outside the circle.

**Figure 7:**
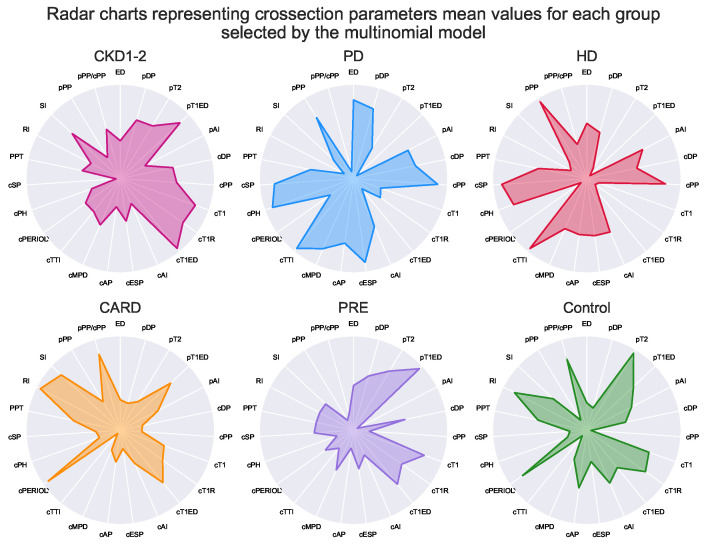
Selected tonometric mean parameters values. The values for each individual parameter were standardized according to the scalar created for the whole patient population (n = 252) and normalized to fall between 0 and 1 values afterward. The radar plots consist of 23 tonometry parameters and show where the means inside every group lie (after scaling). The lowest values are in the circle center, and the highest touch the circumference. The white lines are provided to easily track values calculated for each parameter according to the label outside the circle.

**Figure 8:**
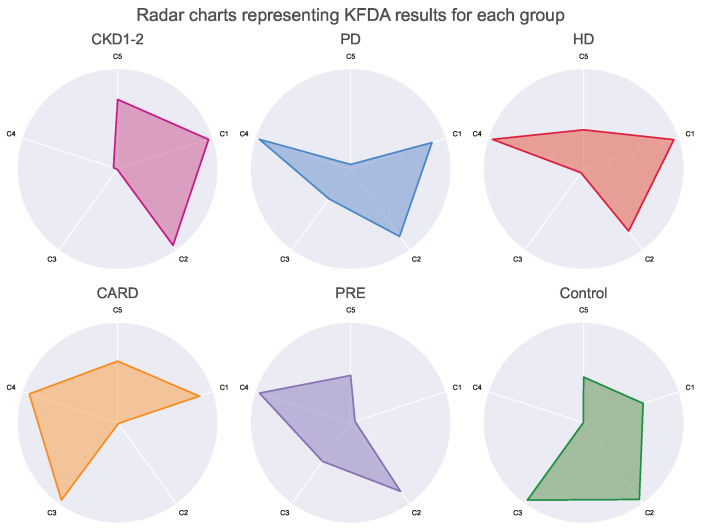
Radar charts for Kernel Fisher Discrimination Analysis results. Each chart represents distinct profiles of the analyzed groups drawn for the mean values of 5 components extracted during dimensionality reduction. The values for each individual component were standardized according to the scalar created for the whole patient population (n = 252) and normalized to fall between 0 and 1 values afterward. The lowest values are in the circle center, and the highest touch the circumference. The white lines are provided to easily track values calculated for each component according to the label outside the circle.

Lastly, Kernel Fisher Discrimination Analysis (KFDA) [11,12] was explored to check whether groups may be the subject of classification. This technique can be regarded as kernelized (allowing nonlinearity), supervised dimensional reduction that maximizes differences between groups and minimizes within them. Among multiple kernels for discrimination analysis: linear, polynomial, laplacian, sigmoid and Gaussian, the laplacian one outperformed the rest in the initial experimental tests (see Table S2). The subset of parameters selected by the multinomial logit model was used as input for discrimination analysis. Detailed results are shown in Figure 5. Additionally, the impact of each step was visualized on three sets of radar chars, first for all parameters (Figure 6), then for the subset of variables selected by the multinomial model (Figure 7), and lastly for the final KFDA result (Figure 8).

Almost every part of the data analysis was performed in Python 3.8. Kruskal–Wallis H tests, $\chi^{2}$ tests, and Spearman ρ correlation coefficients were calculated with scipy (1.3.3) [13]. Correlation networks were analyzed and visualized with the help of NetworkX (2.5) [14]. A multinomial logit model were implemented in RStudio (2021.09.1), with the use of R (4.1.2) and the following libraries: nnet (7.3–17) and MASS (7.3–55) [15], tidyverse (1.3.1) [16]. Discrimination analysis was executed in Python 3.8 with package Kfda (0.1.1) [17] that implements methods described by Ghojogh et al. [12]. The following general purpose and visualization Python packages were used: numpy (1.19.5) [18], pandas (1.2.4) [19,20], matplotlib (3.1.2) [21,22], seaborn (0.11.0) [23] and sci-kit learn (1.0.1) [24].

## 3. Results

### 3.1. General Information

Basic information about the groups is summarized in Table 1. No significant differences in the age of patients were detected between the studied groups (Kruskal–Wallis H = 8.3386, *p* = 0.1386). Sex ratio in each group was similar ($\chi^{2}$ = 6.6365 , *p* = 0.2491) although some variation for percent of tobacco users is present ($\chi^{2}$ = 43.2159, *p* < $10^{-4}$).

Additional information about body mass index (BMI) and percent of overweight patients is presented in Table 2. As may be noted, dialyzed patients (HD and PD groups) possessed lower values of BMI. In contrast, the highest average BMI and percent of overweight patients may be found in CKD1-2 and CARD groups. The overall composition of those group reveal significant differences in both BMI (Kruskal–Wallis H = 66.2484, *p* < $10^{-4}$) and percent of overweight patients ($\chi^{2}$ = 68.9284, *p* < $10^{-4}$).

Crucial information about mortality and duration of dialysis treatment is illustrated in Figure 2. Plot A in Figure 2 shows a strong disproportion in variance and minor mean difference for the number of months during which patients were subjects of dialysis (Kruskal–Wallis H = 11.5238, *p* = 0.0007). This could be a result of treatment accessibility, assignment, and popularity. The highest mortality is seen in group HD reaching almost 30%; a similarly high percentage, close to 25%, is observed for PD and CARD groups (see Figure 2B). No death was recorded for the control group within 2 years of follow-up. regression plot is presented in Plot C within Figure 2. As can be observed, age was not highly correlated with survival (Spearman correlation, ρ = −0.075, *p* = 0.7194) though it was more likely that older patients would die during 2 years of follow-up (Kruskal–Wallis H = 6.6758, *p* = 0.0098).

### 3.2. Differences between the Groups

To detect and analyze differences for each cardiovascular parameter, Kruskal–Wallis tests were performed and are summarized in Table 3. Due to the high number of parameters, z-score plot was chosen to provide an overall illustration of differences in variable values in each group (Figure 3). Additionally, detailed boxplots were created to further illustrate group differences. These figures have been placed in Supplementary File S1 (Figures S1–S7).

As best illustrated by Table 3, the majority of the parameters (34 out of 39) are significantly different in the overall comparison performed by the Kruskal–Wallis test. This might be interpreted as a good initial indicator of the possibility of creating good diagnostic tonometric profiles for every group.

Group differences are well depicted by the simplified z-score plot in Figure 3. This graphic reveals that hemodialyzed patients (HD) and peritoneal dialyzed patients (PD) tend to fall into extremes together. A bit closer to control are groups PRE and CKD1-2. Interestingly, patients without kidney disease but with cardiological issues seem to have the values of the majority of parameters the most similar to the control group. This might indicate that kidney functional disorders may be reflected in a state of the vessel and overall cardiovascular image. Since the z-score plot in Figure 3 presents compressed information, boxplots with more details have been added to Supplementary File S1 (see Figures S1–S7).

### 3.3. Correlation Structure of the Groups

Tonometric parameters are expected to be correlated. Since most of the analyzed variables are signal-related features or stay in a direct relationship or dependence from each other, it is reasonable to anticipate a high number of strong correlations. To demonstrate how codependence of parameters is influenced by the presence of cardiovascular distortions, an effort was made to create correlation graph profiles for each group analyzed in this study.

Spearman correlations for each pair of variables were performed separately for each group. Detailed correlation heatmaps are presented in Supplementary File S1 (Figures S8–S13). In the case of the analysis of correlations, the focus was kept on general trends rather than individual relations between variables. Although many individual corrections are strong and significant, overall trends easily emerge from the results.

Figure 4 visualizes strong and significant Spearman correlation (ρ≥0.7 and p<0.01) in the form of a relation graph for each group. The number of edges that denote correlations is the lowest for the control group and relatively strongly elevated for the others. Patients with cardiovascular and/or kidney problems correspond to graphs more densely filled with connections, sometimes forming only one component that seems to drift towards a complete graph, where each node is connected to every other one. Although patterns within the groups are not identical, this effect could be caused by pathological changes that are responsible for a cardiovascular profile, where most of the tonometric parameters are altered.

Summarized information about correlation graphs has been placed in Table 4. As it may be seen, surplus (rarely depleted) correlations are often associated with pSP, pDP, pMEANP, cDP, cTTI, cSP, RI, and pPP. All of the mentioned parameters strongly diverged between the groups. Correlation differences in various groups may reveal distinct features of altered vessels in each group.

Although differences in correlations may seem interesting, parameters that have little or no associations with others can turn out to be the most significant to the profiles. In fact, such parameters may hold unique information about patients’ conditions that in case of dense correlation could be easily overlooked.

### 3.4. Dimensionality Reduction and Profile Creation

Assuming that tonometric cardiovascular parameters are capable of good characterization of the groups analyzed in this study, an effort was made to extract the simplest possible profiles for each group. To do this, multistep analysis was performed.

At first, the best model was chosen to filter out some less significant parameters, i.e., a multinomial logistic regression model was developed in R based on an AIC step-wise approach. The results of this analysis indicated that 23 out of 39 parameters are important for the model. The results are summarized in Supplementary File S1 (Table S1). Since the model is overcomplicated and serves as a filter technique, further details are irrelevant and shall be omitted.

The multinomial model was later treated as a feature selection method along with mean and variance analysis and correlation graph analysis. Six sets of parameters were compared as input for the discrimination analysis (see methods in Section 2.4 for details). A multinomial model was found to be the most effective in the separation of the groups (with accuracy equal to 86.81% patients were differentiated into consistent groups according to an original label). Therefore, a set of parameters proposed by this method was further analyzed and visualized in this study. More details, results, and selection criteria are summarized in Table 5. Moreover, in Supplementary File S1 (Table S2), information about KFDA kernels comparison is presented.

Kernelized discrimination analysis is a nonlinear version of linear discrimination analysis. The effectiveness of this method used on tonometric data may suggest that differences between the groups may have a nonlinear nature. However, techniques with mean and variance analysis and selection of poorly correlated parameters were able to boost the accuracy.

Moreover, KFDA returns similarly good results for tested combinations of parameters. As it may be believed, this could be the effect of many strong correlations between the variables. In fact, that was the reason for using the multinomial logistic regression model in the first place. The result of the multinomial model contains a subset of variables that together have the ability to characterize each group sufficiently. Notice that the high number of correlations actually implies that there are many other similar subsets of variables able to perform as well as discrimination analysis input chosen by us. Our subset is only a proposition that was obtained by step-wise selection of a regression model based on AIC. It is also worth noting that a combination of parameters with few correlations had the second-best result. Therefore, a simpler method of correlation analysis may quickly reveal interesting parameters. Since the total number of parameter subsets is $2^{39}$, exploration of all possibilities would be tedious (and, in fact, impossible) and, in our opinion, unnecessary.

On the one hand, tonometric parameters taken together may reveal a good profile specific to cardiovascular conditions and kidney problems. On the other hand, relations that differentiate the groups are nonlinear. Moreover, the data is largely redundant due to the structure and the high number of correlations. Hence, compression completed by dimensionality reduction seems to be natural.

A visualization of KFDA results is illustrated in Figure 5 in the form of 3D scatterplot. It is important to note that every group was amazingly well separated by KFDA, except for HD and PD. This makes sense since differences between hemodialyzed and peritoneal dialyzed patients in tonometric parameters or cardiovascular states are not expected by default. As can be spotted, dialysis patients have similar profiles (Figure 6, Figure 7 and Figure 8), unlike the other groups.

The whole dimensionality reduction process was illustrated by three sets of radar charts. The full set of parameters is shown in Figure 6. The subset of parameters that was obtained from the results of the multinomial logistic model is shown in Figure 7. Finally, the results of KFDA are depicted on charts framed together in Figure 8.

The performed discrimination analysis yielded five different components, which are dimensionality reduction results. Each component describes differences between set parameters variance from a different angle. As shown in Figure 8, the means of the components form a unique pattern for each group. Profiles created by this method are strong since there is a small variance between patients within the same group and strong differences between the groups; all five components were considered.

Although the presented radar plots do not show exact values, their aim was to visualize all parameters at once and depict overall group profiles. Based on such plots, it is possible to see how similar the groups are on the higher level without focusing on single parameters. On the one hand, this visualization method may seem imprecise and oversimplifying (notice that all the patients’ variance within a group was compressed to a single point representing the mean). However, it is difficult to capture general patterns for many parameters simultaneously, and radar plots appear to be surprisingly clear in the presentation of high-level profiles. For example, plots in Figure 6 for HD and PD groups appear to show some intuitive resemblance, being at the same time distinct from the image representing the CARD profile.

## 4. Discussion

In this study, multiple approaches were used to analyze the internal structure and vascular profiles based solely on tonometric data collected from patients with cardiovascular and kidney disorders. It can be concluded that there is no single tonometric parameter able to sufficiently separate each of the six analyzed groups (HD, PD, CARD, PRE, CKD1-2, and Control). Moreover, proportionally large subsets of parameters were highly correlated in each group, although exact correlations seem to be different within groups. It should be pointed out that the healthy subjects seem to have the smallest number of high correlations between parameters. The graphs with correlations created for each group may have good potential for differentiation between the groups.

Some light is shed by the results of the multinomial regression model, where more than a half (23/39) of parameters were included in the best model by the step-wise method (many similar models may be created due to the high number of correlations) that was used in this study as a feature selection technique. It could be suggested that either the model takes advantage of the noisy variance in the data or the search for linear models to separate the groups is fruitless and the key information about the unique traits of each group cannot be attributed to parameters themselves but to the relations between them.

Kernel Fisher Discrimination Analysis results seem to support the second scenario. Nonetheless, at this moment , it should be pointed out that deriving conclusions from the data must be carefully considered, and more research is needed to further support this interpretation of the results. KFDA is a nonlinear kernel-based method for class separation [12]. Since it is possible to create good profiles distinguishing all groups with KFDA, creating a model that classifies stages of kidney disease should be possible based on tonometry data. The creation of such a model would be possible if more data were available. At this stage, based on the obtained results, it can be suggested that machine learning has great potential in this case. Alternatively, it could be hypothesized that a set of simple nonlinear equations exists that denote relations between tonometric variables that will separate each group well. Since each parameter corresponds to a different aspect of a tonometric signal shape, a detailed exploration of morphological aspects of the data would reveal a practical set of unique signal characteristics that could be used in diagnostics, monitoring, and screening for CKD as well as analysis of hemodialysis treatment impact on patient arterial and overall condition.

Fe-fusion and dimensionality reduction methods are still being developed, and some new techniques are worth mentioning as they have great potential for future use. For example, discrimination power analysis is a promising technique that can create precise individual profiles for identification purposes [25]. Other popular methods include supervised principal component analysis and negative matrix factorization; both could be successfully used for profile creation (see [26,27] as examples). Modern data set visualization techniques or manifold learning methods are very popular, e.g., the use of t-distributed stochastic neighbor embedding on transcriptomic [28] or spectral embedding fusion on methylation [29] single-cell data. All of these methods have great potential and could be used on a bigger data set to create models usable in medical practice.

Creating machine learning models based on tonometry results was completed several times. Kublanov et al. [30] compared many different techniques (linear and quadratic discriminant analysis, k-nearest neighbors, support Vector machines, decision trees, and naïve Bayes classification) to classify control and hypertension patients based on short-term rate variability signals that contributed to 53 tonometric features. The paper found discriminant analysis to be the best approach. However , the work itself had strong methodological issues, namely the small sample size (30 and 40 patients in control and hypertension groups, respectively [30]).

In fact, group sizes in this study are not sufficient to create a useful machine learning model either (see Table 1). That was the reason for choosing predominantly statistical techniques (correlations, non-parametric variance tests). Only generalized linear models and KFDA can be regarded as machine learning techniques. Still, for this case, the statistical analysis which was performed serves as a good base for KFDA results interpretation. Moreover, it is explicitly stated that more data are needed to support promising results in this study.

Another example of the use of machine learning on hemodynamics data was provided by Jones et al. [31]. The authors classified and detected different forms of stenosis and aneurysms; in this case, data describing pathological states were created artificially based on theoretical models and many measurements for healthy patients.

Garcia-Carretero and others found that arterial stiffness evaluated by pulse wave velocity features turns out to be a good predictor of cardiovascular disease development [32]. The authors used Cox regression and proved pulse wave velocity to be a good predictor of future cardiac problems within 12.4 months follow-up in the prediabetic population. Almeida and others proposed a framework to analyze arterial pressure waveforms with machine learning algorithms [33]. Despite a small number of subjects (50 divided into 3 groups), the researchers tried to classify control subjects and hypertensive patients based on a combination of multiple algorithms, that is random forest, BayesNet, J48 and RIPPER entangled together in the majority-voting system. The researchers showed that non-invasive techniques combined with advanced data processing and analysis can serve as cheap and reliable predictors of cardiovascular system health [33].

Nabeel et al. used an artificial neural network as a method for continuous blood pressure estimation [34]. To build a deep learning model, the authors used an image-free ultrasound system and based it on multiple collected variables for arterial luminal diameter waveforms taken from 20 subjects and demonstrated in in vivo experiments that this non-invasive technique has promising clinical potential. Another example of machine-learning-based blood pressure estimation based on pulse waveform was performed by Chen et al. [35]. This analysis took data from the MIMIC-III Waveform Database, which stores data from critical care units [36]. The researchers used the mean impact value to filter out unpromising data dimensions. Support vector regression was used to estimate blood pressure with parameters optimized by a genetic algorithm [35]. In fact, there is intensive research in machine learning techniques for blood pressure estimation based on data collected in a non-invasive manner (for more, see: [37]).

CKD has impact on arterial stiffness [38]. The tonometry data collected from patients with CKD need to be estimated differently than the data taken from other patients. It was shown for central blood pressure that different progression stages of CKD require additional calibration to be correct [9].

It is worth mentioning that tonometers may become easily accessible devices, including smartphones that may have some of their functions. In fact, Wu et al. [39] developed a smartphone-based tonometer. One can imagine additional software components that may theoretically predict cardiovascular diseases and give more information about chronic kidney disease state and progression. The potential is huge, including the ability to predict the likelihood of risks that may be a consequence of kidney malfunctioning. Moreover, all of this could be performed by the patient himself/herself at home. Bodington and coauthors reviewed recent new technologies that may help patients with CKD and be used at home [40].

It should be noted that arterial stiffness is one of the earliest symptoms of cardiovascular dysfunction in CKD. It can be detected even before an overt impairment of the ejection fraction or diastolic filling. The gold standard for measuring aortic stiffness is invasive aortic catheterization; however, it is an expensive, risky, and clinically impractical study. Hence, earlier and improved quantification of arterial stiffness in patients may improve disease risk stratification and is an attractive imaging biomarker for use in clinical trials. Hence, the use of non-invasive methods of aortic stiffness assessment in patients with CKD, ESRD, and CVD has great potential.

## 5. Conclusions

The importance of screening and prevention in medicine has always been highlighted. Non-invasive vascular tonometry is a typical examination that helps in everyday diagnostics of CVD patients. This study shows that it is possible to assess kidney condition along typical tonometry measurements. Although the set of data analyzed in this study is too small to create an accurate machine learning model that could be validated and applied in clinical reality, clear patterns and distinct profiles of CKD patients are revealed.

In recent years progress in machine learning and statistical tools made extraction and validation of complex features easier. Thanks to advancements in methods and software, it was possible to perform multiple analyses (analysis of variance, network correlation structure analysis, multinomial logistic regression, and discrimination analysis) on tonometric data and create profiles for each of the examined groups. The advanced data analysis points out that deploying a model that would differentiate between groups is possible. Such a model could be used as a CKD screening tool during aregular vascular tonometric assessment. Although it is probably possible to create a complex feature reflecting kidney condition, the true relationships within the data are likely nonlinear and may be difficult to model by simple mathematical expressions. It is also worth noting that more advanced examinations such as 3D vessel reconstruction [41] could shed more light on arterial profiles of patients from different cohorts.

Tonometry may disclose a detailed vascular profile which is particularly important for CKD patients, as arterial hypertension and CKD are often strongly connected. It is well-known that the condition of kidneys depends on cardiovascular health and vice versa. Since CKD is a global problem that affects 10% of the worldwide population [42], faster diagnosis may improve prognosis and life expectancy of people with hidden kidney dysfunction. This study shows that simple tonometric examination has high screening potential and can be used as a tool for monitoring CKD development.

## Figures and Tables

**Table 1:** Basic information about age (mean ± standard deviation), sex (percent of females), and tobacco usage (percent of smokers) within the groups.

	Age	Sex (% Female)	Smoking
HD (n = 71)	57.51 ± 14.84	32.4%	21.13%
PD (n = 35)	52.83 ± 18.33	42.86%	0%
PRE (n = 48)	62.64 ± 10.72	47.92%	12.5%
CARD (n = 37)	59.84 ± 8.33	32.43%	18.92%
CKD1-2 (n = 29)	60.93 ± 12.04	34.48%	0%
Control (n = 32)	60.28 ± 12.49	46.87%	6.25%

**Table 2:** Basic information about body mass index (mean ± standard deviation) and obesity (percent of overweight patients) within the groups.

	BMI	% Overweight
HD (n = 71)	22.96 ± 3.67	21.12%
PD (n = 35)	23.41 ± 3.22	28.57%
PRE (n = 48)	25.45 ± 3.66	50%
CARD (n = 37)	28.16 ± 4.1	72.97%
CKD1-2 (n = 29)	28.19 ± 3.06	86.21%
Control (n = 32)	25.04 ± 4.05	34.37%

**Table 3:** Differences between groups presented as results of the Kruskal–Wallis H test for each cardiovascular parameter analyzed. The critical value of H statistic is equal to 11.07. Strong differences are marked with bold text.

Parameter Name	H	*p*-Value
**ED**	**20.5221**	**0.001**
**pSP**	**52.609**	<$10^{-4}$
**pDP**	**29.0132**	<$10^{-4}$
**pMEANP**	**58.639**	<$10^{-4}$
**pT1**	**22.7532**	**0.0004**
**pT2**	**22.2066**	**0.0005**
**pP1**	**62.9692**	<$10^{-4}$
**pP2**	**57.3636**	<$10^{-4}$
**pT1ED**	**22.2034**	**0.0005**
**pT2ED**	**42.1697**	<$10^{-4}$
pAI	9.9668	0.0762
**pESP**	**36.9327**	<$10^{-4}$
**cMEANP**	**48.0839**	<$10^{-4}$
**cPP**	**62.1512**	<$10^{-4}$
**cT1**	**17.679**	**0.0034**
**cT2**	**23.2157**	**0.0003**
**cT1R**	**13.8238**	**0.0168**
**cP1**	**52.908**	<$10^{-4}$
**cP2**	**52.375**	<$10^{-4}$
**cT1ED**	**15.3751**	**0.0089**
cT2ED	9.7243	0.0834
**cAI**	**13.7502**	**0.0173**
**cESP**	**42.3102**	<$10^{-4}$
**cAP**	**27.3468**	<$10^{-4}$
**cMPS**	**53.2218**	<$10^{-4}$
**cMPD**	**33.5388**	<$10^{-4}$
**cTTI**	**85.9245**	<$10^{-4}$
cDTI	5.0134	0.4142
**cSVI**	**57.7267**	<$10^{-4}$
**cPERIOD**	**25.2241**	**0.0001**
**cDD**	**37.1145**	<$10^{-4}$
**cPH**	**46.1326**	<$10^{-4}$
**cSP**	**63.5783**	<$10^{-4}$
**cDP**	**20.907**	**0.0008**
PPT	10.2408	0.0687
**RI**	**20.1941**	**0.0011**
SI	5.8177	0.3244
**pPP**	**53.8578**	<$10^{-4}$
**pPP/cPP**	**23.6809**	**0.0003**

**Table 4:** Node degree distribution in correlation graphs with additional $\chi^{2}$ test results for every parameter. Strong differences are marked with bold text.

Parameter Name	Control	HD	PD	PRE	CARD	CKD1-2	$\chi^{2}$	*p*-Value
ED	0	1	4	2	4	2	5.9231	0.3138
**pSP**	**0**	**15**	**11**	**16**	**13**	**15**	**15.3714**	**0.0089**
**pDP**	**1**	**14**	**5**	**13**	**13**	**12**	**14.8276**	**0.0111**
**pMEANP**	**1**	**15**	**12**	**14**	**13**	**14**	**11.9565**	**0.0354**
pT1	1	1	3	1	1	1	2.5	0.7765
pT2	0	0	4	1	2	1	8.5	0.1307
pP1	12	15	11	15	13	15	1.1481	0.9498
pP2	11	16	14	18	12	12	2.6627	0.7518
pT1ED	1	3	5	1	1	1	7	0.2206
pT2ED	0	1	0	1	1	0	10.3333	0.0663
pAI	2	2	2	6	0	4	8	0.1562
pESP	9	14	7	16	13	15	5.1351	0.3996
**cDP**	**3**	**6**	**2**	**14**	**3**	**9**	**17.3243**	**0.0039**
cMEANP	10	15	12	14	13	14	1.2308	0.9419
cPP	2	6	8	7	4	1	8.4286	0.1341
cT1	3	3	4	2	1	2	2.2	0.8208
cT2	0	2	3	3	4	3	3.8	0.5786
cT1R	3	1	2	2	1	2	1.5455	0.9078
cP1	12	15	13	14	14	14	0.3902	0.9956
cP2	11	17	13	17	13	17	2.4091	0.7901
cT1ED	3	2	4	2	2	2	1.4	0.9243
cT2ED	0	0	2	3	0	4	3	0.7
cAI	6	3	3	6	4	5	2.1111	0.8336
cESP	10	16	16	15	13	15	1.8941	0.8636
cAP	4	4	5	6	4	5	0.7143	0.9822
cMPS	12	14	12	14	13	16	0.8519	0.9736
cMPD	11	14	10	14	13	13	1.08	0.9559
**cTTI**	**7**	**11**	**0**	**1**	**13**	**5**	**22.1892**	**0.0005**
cDTI	7	12	8	14	12	13	3.6364	0.6029
cSVI	1	3	4	2	1	2	3.1538	0.6763
cPERIOD	1	3	4	2	2	2	2.2857	0.8084
cDD	2	2	5	2	1	2	4	0.5494
cPH	3	7	8	7	4	7	3.3333	0.6487
**cSP**	**11**	**14**	**10**	**16**	**1**	**14**	**13.0909**	**0.0225**
PPT	1	1	3	2	2	0	3.6667	0.5983
**RI**	**0**	**0**	**5**	**2**	**2**	**0**	**13**	**0.0234**
SI	1	1	1	2	2	0	2.4286	0.7872
**pPP**	**6**	**7**	**8**	**1**	**0**	**0**	**18.9091**	**0.002**
pPP/cPP	0	2	1	2	2	1	2.5	0.7765
Sum	168	278	244	290	230	260		

**Table 5:** Comparison of different approaches for feature selection based on evaluated mean accuracy of the KFDA model. Additional information about a number of chosen parameters, selection criteria, and methods used is given in distinct columns. The best feature selection method was highlighted with bold text.

Approach	Chosen Method	Selection Criteria	Number of Selected Parameters	KFDA Accuracy
No selection	None	None	39	0.8 ± 0.083
Mean and variance analysis	Standardized means visualization and Kruskal–Wallis test	H ≥ 46 and *p* $<10^{-4}$ with additional clear pattern on the standardized means plot.	14	0.8184 ± 0.0524
Correlation graph analysis	Spearman correlation test	Significant ($\chi^{2}$ test) differences between node degrees on correlation graphs.	8	0.7763 ± 0.0602
Correlation graph analysis	Spearman correlation test	Nodes with no more then 10 correlation within each group.	23	0.8462 ± 0.1027
**Generalized** **linear model**	**MNLogit**	**Step-wise search**	**23**	**0.8681** ± **0.0636**
Mixed	Set intersection	Common parameters that can be found both in results of MNLogit and list of variables with few correlations.	17	0.7921 ± 0.051

## Data Availability

All necessary data are included in the paper.

## Supplementary Materials

The following are available online at https://www.mdpi.com/article/10.3390/ijerph191912339/s1, Figure S1: Boxplot depicting differences between HD, PD, PRE, CARD, CKD1-2 and Control for chosen variables: ED, pSP, pDP, pMEANP, pT1 and pT2; Figure S2: Boxplot depicting differences between HD PD, PRE, CARD, CKD1-2 and Control for chosen variables: pP1, pP2, pT1ED, pT2ED, pAI and pESP; Figure S3: Boxplot depicting differences between HD, PD, PRE, CARD, CKD1-2 and Control for chosen variables: cDP, cMEANP, cPP, cT1, cT2 and cT1R; Figure S4: Boxplot depicting differences between HD, PD, PRE, CARD, CKD1-2 and Control for chosen variables: cP1, cP2, cT1ED, cT2ED, cAI and cESP; Figure S5: Boxplot depicting differences between HD, PD, PRE, CARD, CKD1-2 and Control for chosen variables: cAP, cMPS, cMPD, cTTI, CDTI and cSVI; Figure S6: Boxplot depicting differences between HD, PD, PRE, CARD, CKD1-2 and Control for chosen variables: cPERIOD, cDD, cPH, cSP, PPT and RI; Figure S7: Boxplot depicting differences between HD, PD, PRE, CARD, CKD1-2 and Control for chosen variables: SI, pPP and pPP/cPP; Figure S8: Correlation heatmap for all possible pairs of tonometric parameters within the Control group; Figure S9: Correlation heatmap for all possible pairs of tonometric parameters within the HD group; Figure S10: Correlation heatmap for all possible pairs of tonometric parameters within the PD group; Figure S11: Correlation heatmap for all possible pairs of tonometric parameters within the PRE group; Figure S12: Correlation heatmap for all possible pairs of tonometric parameters within the CARD group; Figure S13: Correlation heatmap for all possible pairs of tonometric parameters within the CKD1-2 group; Table S1: Wald test results for the chosen combination of tonometry variables in the multinomial logistic regression model; Table S2: Comparison of different available kernels for KFDA algorithm; Table S3: Group specific descriptive statisctis calculated for tonometry parameters and general biometric data; Table S4: Full Spearman cross correlation results for each group; Table S5: Exact number of patients within each group with additional number of deceased within 2 years follow-up.
