# Supplementary material for: Chronic Kidney Disease as a Cardiovascular Disorder—Tonometry Data Analyses
Source: Int J Environ Res Public Health. 2022 Sep 28;19(19):12339. doi: 10.3390/ijerph191912339 (PMC9566812; doi:10.3390/ijerph191912339)
Supplement: Supplementary file 1 [file ijerph-19-12339-s001.zip › Supplementary Figures S1-S13 & Tables S1 and S2.pdf]

Differences between groups for selected variables (1)

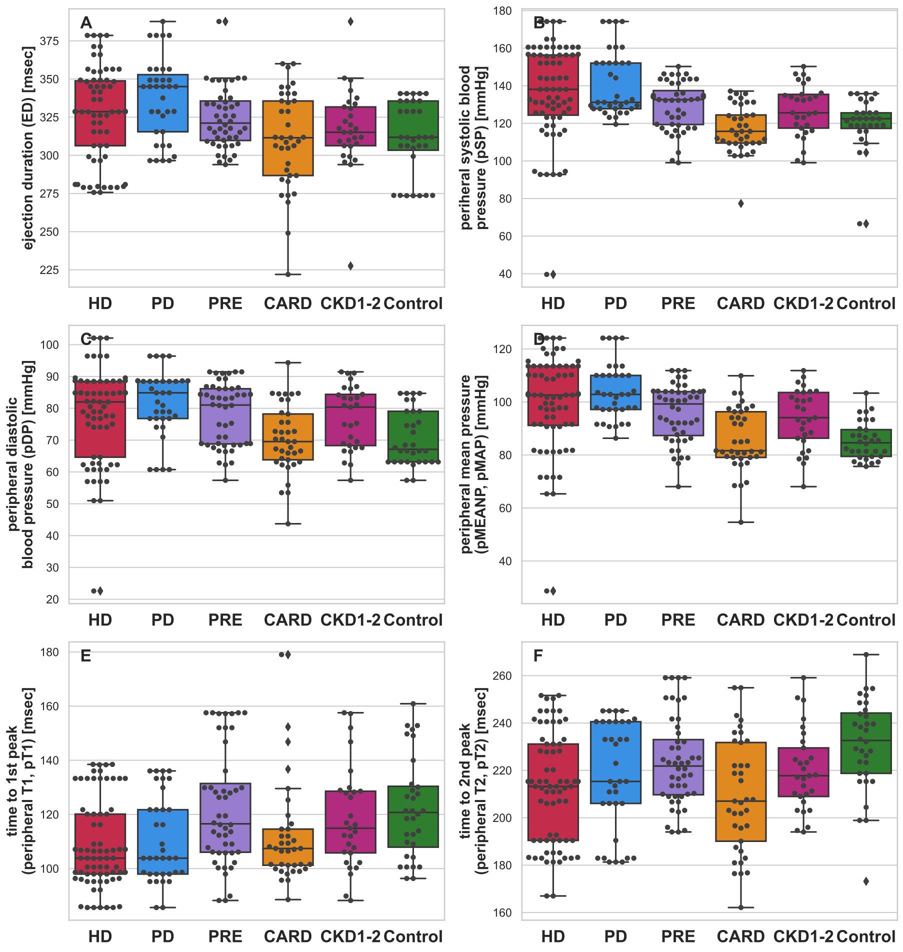

Figure S1: Boxplot depicting differences between HD (red), PD (blue), PRE (purple), CARD (orange), CKD1-2 (pink) and Control (green) for chosen variables : ED (**A**), pSP (**B**), pDP (**C**), pMEANP (**D**), pT1 (**E**) and pT2 (**F**). An additional swarmplot was overlaid on the original plot to mark the obtained measurements for each patient (dots).

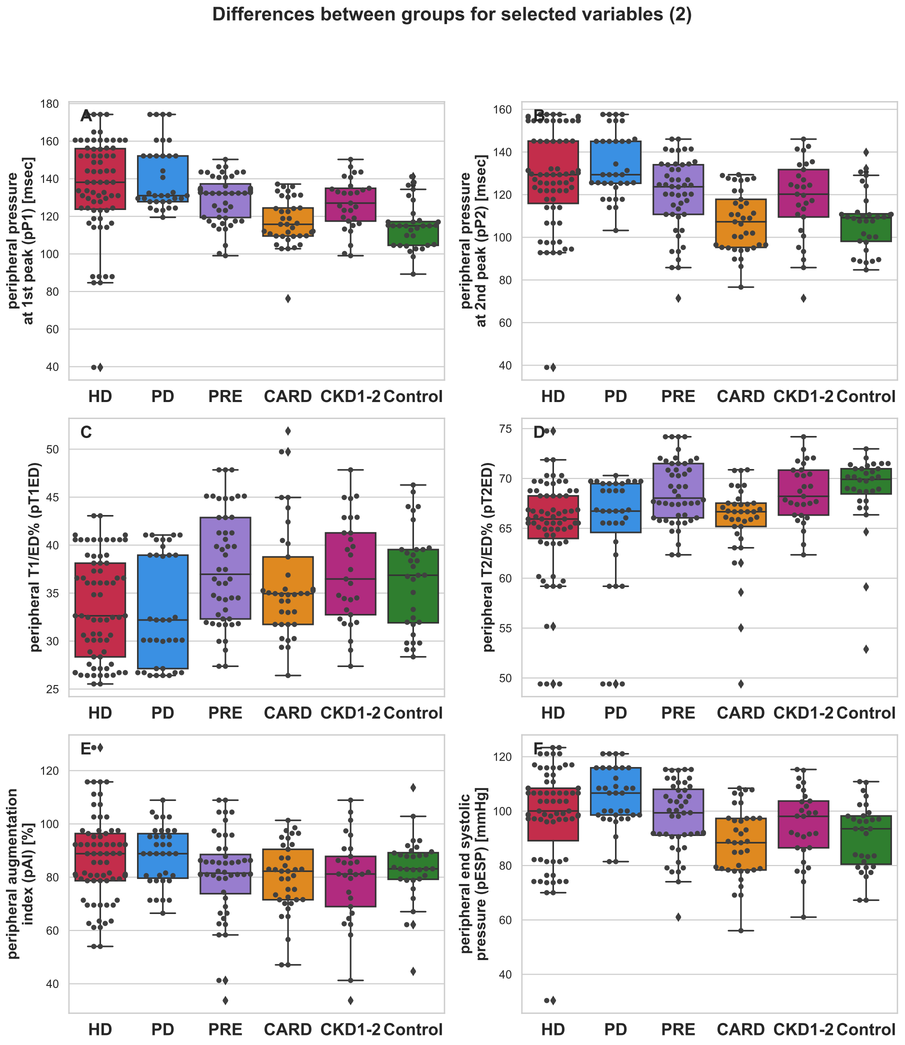

Figure S2: Boxplot depicting differences between HD (red), PD (blue), PRE (purple), CARD (orange), CKD1-2 (pink) and Control (green) for chosen variables : pP1 (**A**), pP2 (**B**), pT1ED (**C**), pT2ED (**D**), pAI (**E**) and pESP (**F**). An additional swarmplot was overlaid on the original plot to mark the obtained measurements for each patient (dots).

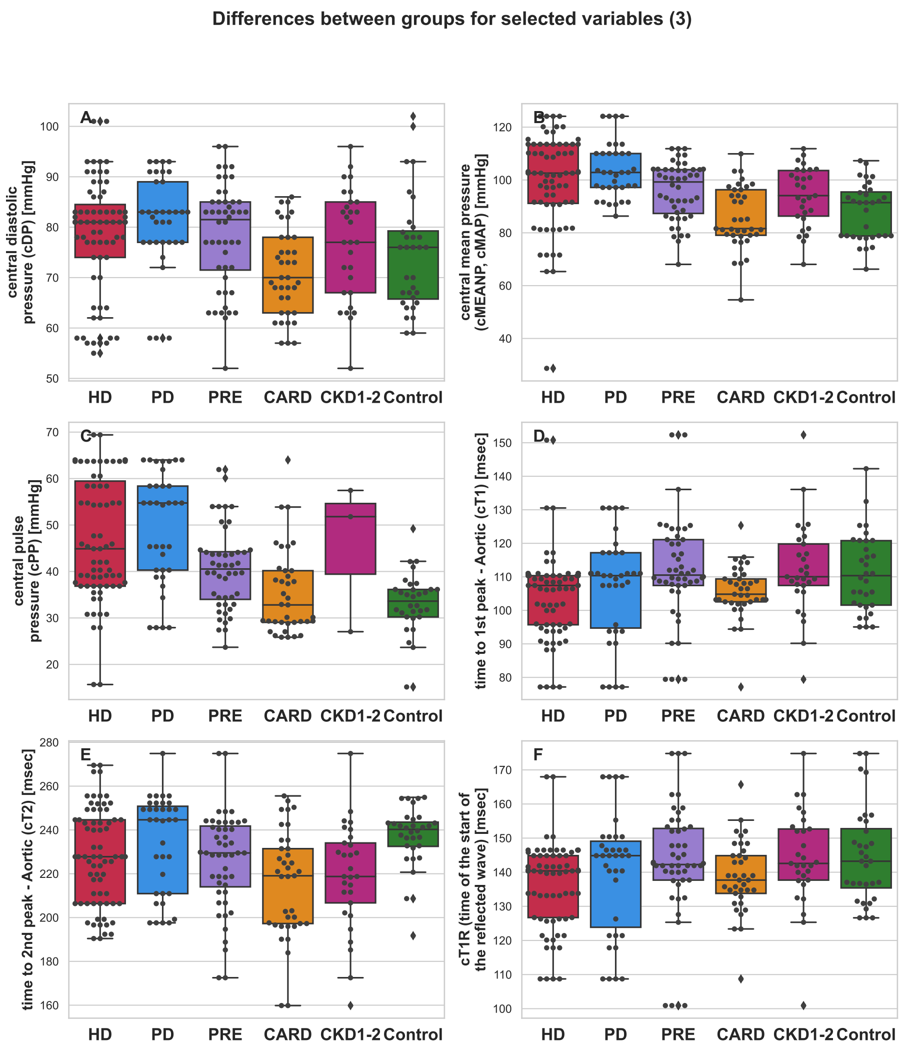

Figure S3: Boxplot depicting differences between HD (red), PD (blue), PRE (purple), CARD (orange), CKD1-2 (pink) and Control (green) for chosen variables : cDP (**A**), cMEANP (**B**), cPP (**C**), cT1 (**D**), cT2 (**E**) and cT1R (**F**). An additional swarmplot was overlaid on the original plot to mark the obtained measurements for each patient (dots).

Differences between groups for selected variables (4)

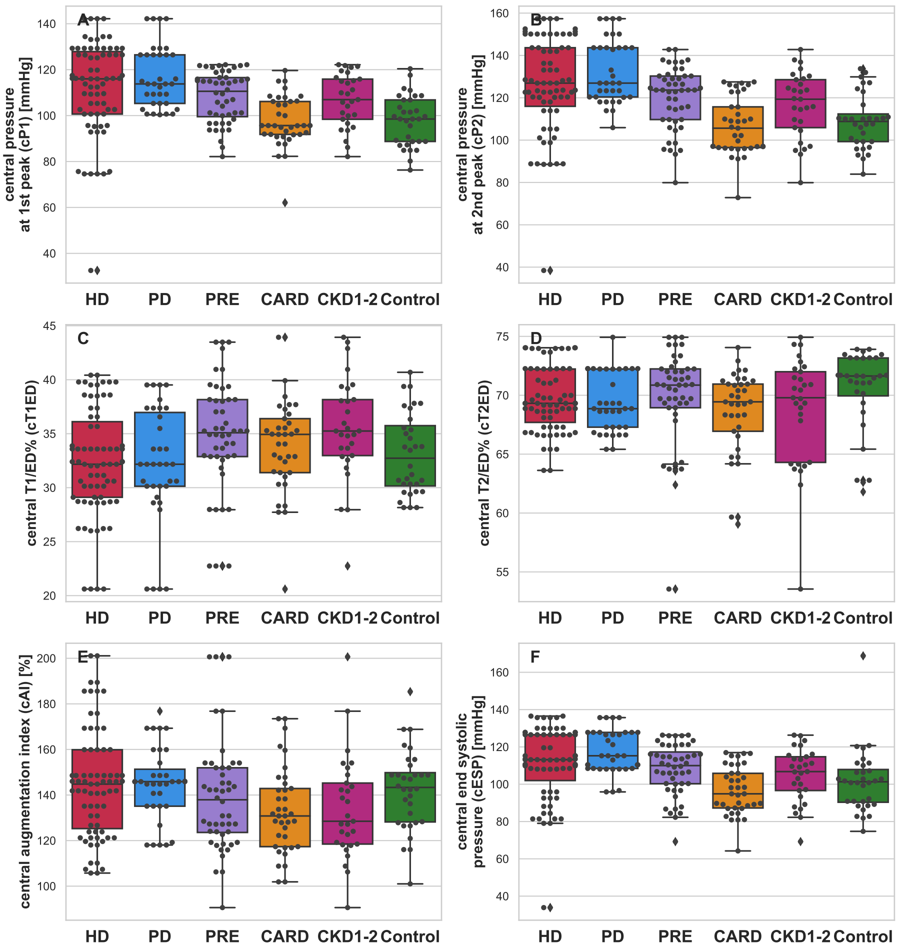

Figure S4: Boxplot depicting differences between HD (red), PD (blue), PRE (purple), CARD (orange), CKD1-2 (pink) and Control (green) for chosen variables : cP1 (**A**), cP2 (**B**), cT1ED (**C**), cT2ED (**D**), cAI (**E**) and cESP (**F**). An additional swarmplot was overlaid on the original plot to mark the obtained measurements for each patient (dots).

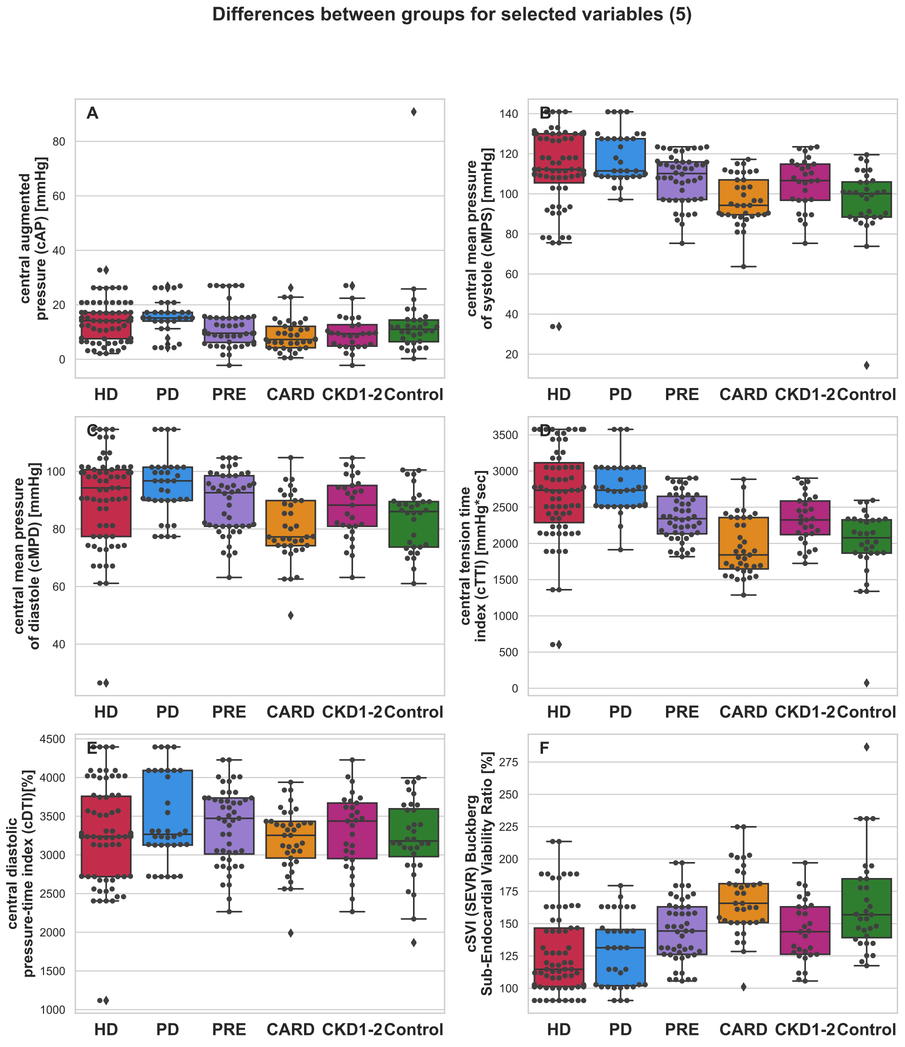

Figure S5: Boxplot depicting differences between HD (red), PD (blue), PRE (purple), CARD (orange), CKD1-2 (pink) and Control (green) for chosen variables : cAP (**A**), cMPS (**B**), cMPD (**C**), cTTI (**D**), CDTI (**E**) and cSVI (**F**). An additional swarmplot was overlaid on the original plot to mark the obtained measurements for each patient (dots).

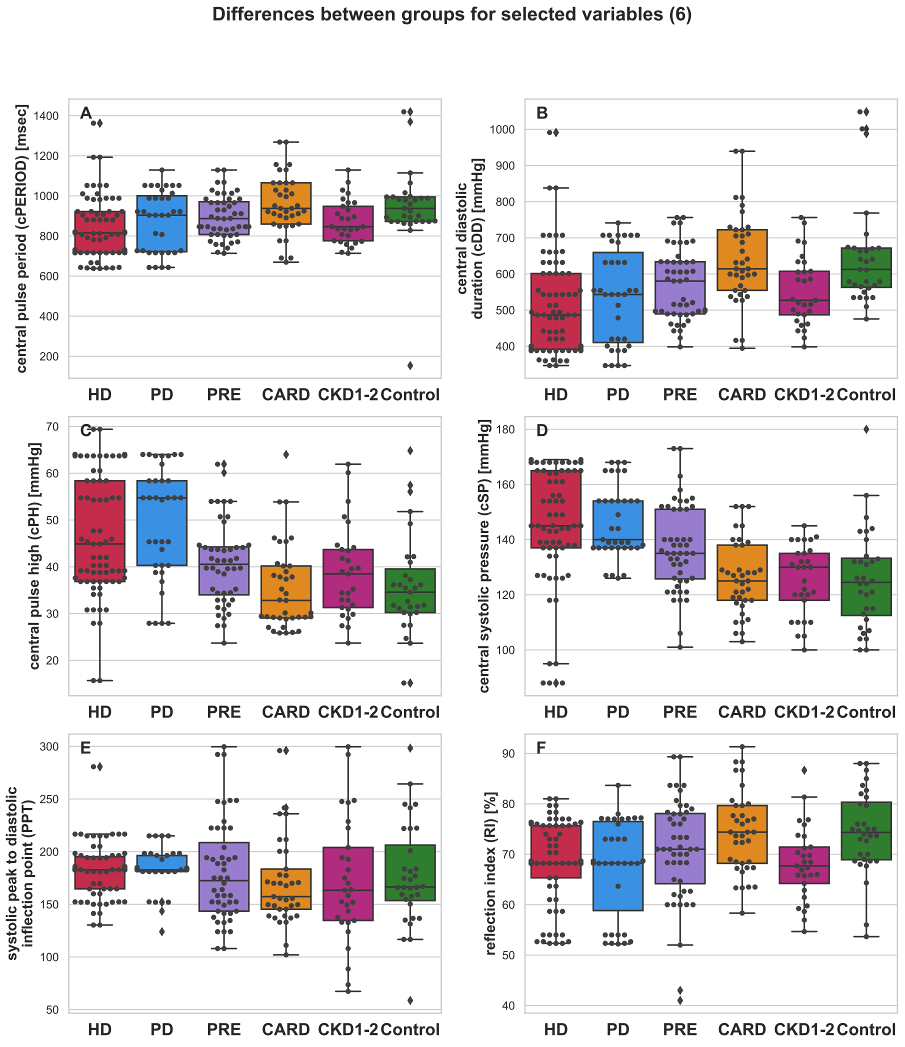

Figure S6: Boxplot depicting differences between HD (red), PD (blue), PRE (purple), CARD (orange), CKD1-2 (pink) and Control (green) for chosen variables : cPERIOD (**A**), cDD (**B**), cPH (**C**), cSP (**D**), PPT (**E**) and RI (**F**). An additional swarmplot was overlaid on the original plot in order to mark the obtained measurements for each patient (dots).

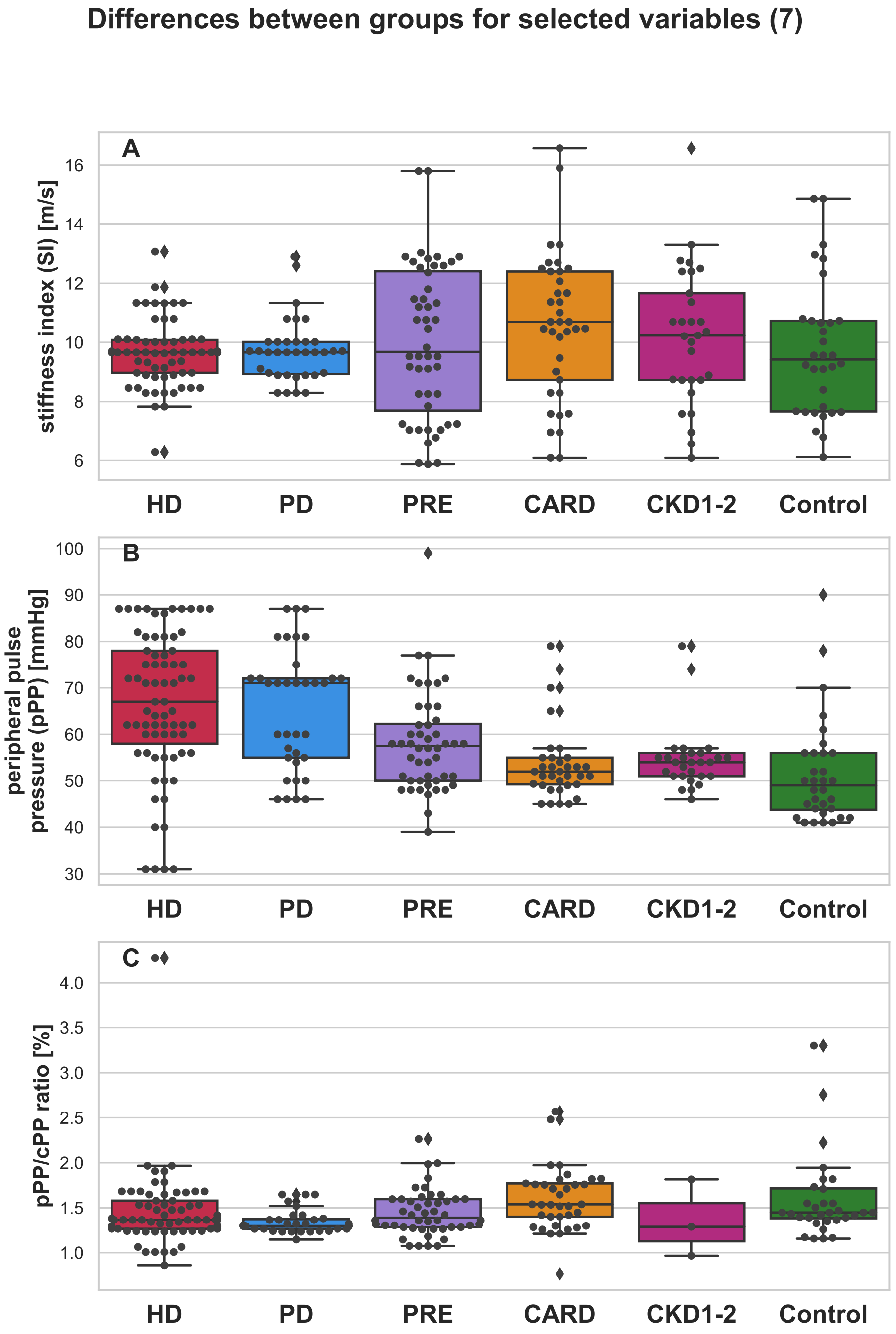

Figure S7: Boxplot depicting differences between HD (red), PD (blue), PRE (purple), CARD (orange), CKD1-2 (pink) and Control (green) for chosen variables : SI (**A**), pPP (**B**) and pPP/cPP (**C**). An additional swarmplot was overlaid on the original plot to the obtained measurements for each patient (dots).

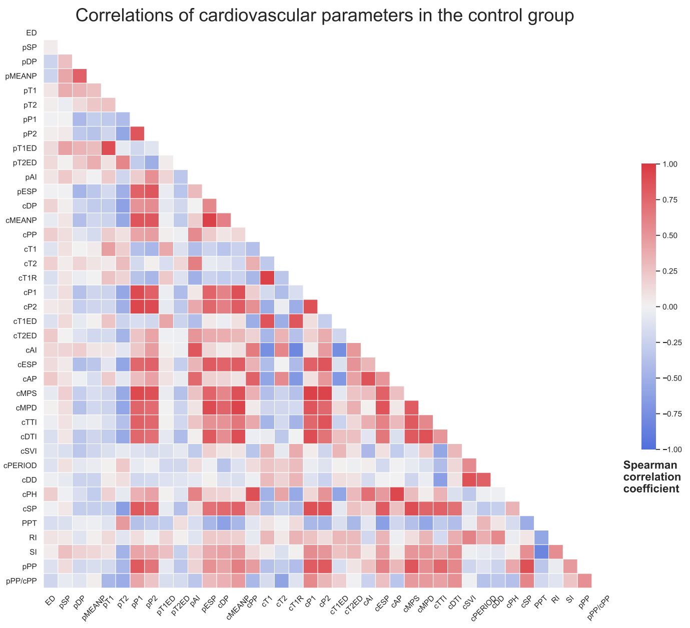

Figure S8: Correlation heatmap for all possible pairs of tonometric parameters within the Control group. The colors indicate values of Spearman correlation coefficient (blue for negative correlation, white for no correlation, and red for the positive relationship).

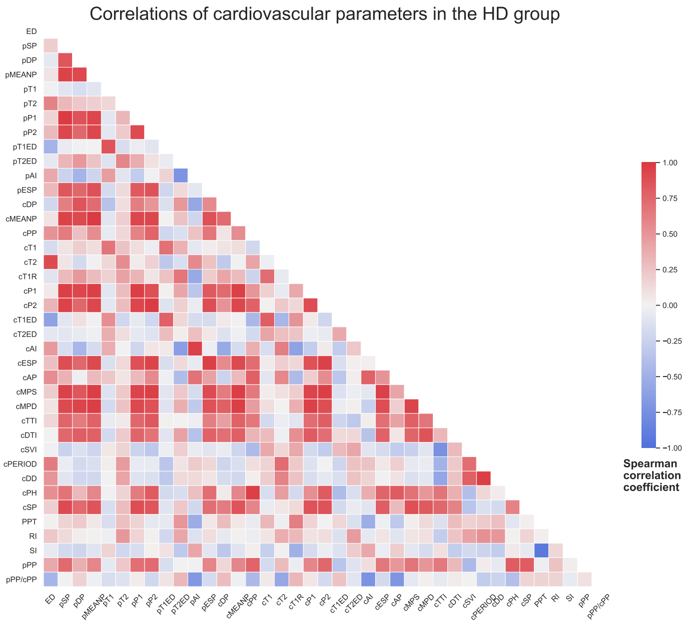

Figure S9: Correlation heatmap for all possible pairs of tonometric parameters within the HD group. The colors indicate values of Spearman correlation coefficient (blue for negative correlation, white for no correlation, and red for the positive relationship).

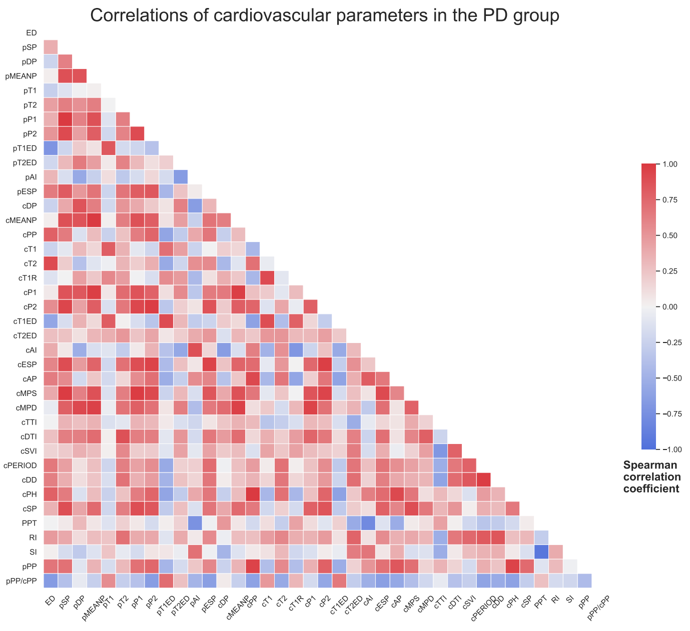

Figure S10: Correlation heatmap for all possible pairs of tonometric parameters within the PD group. The colors indicate values of Spearman correlation coefficient (blue for negative correlation, white for no correlation, and red for the positive relationship).

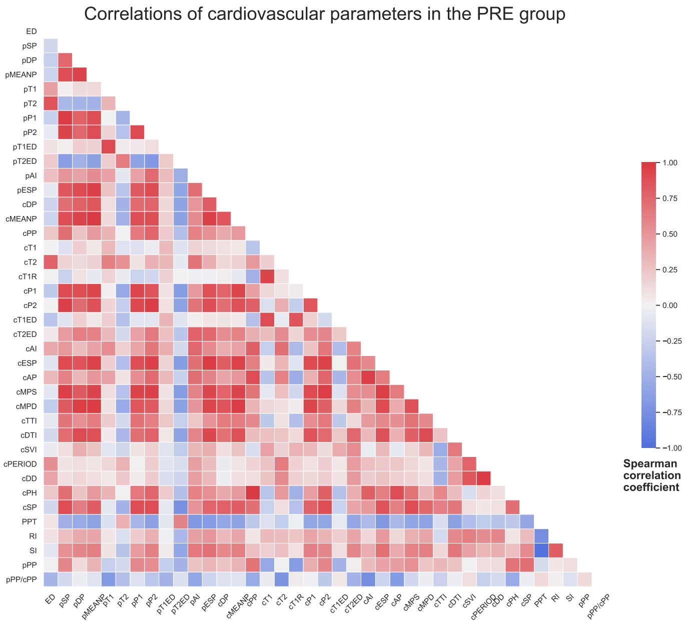

Figure S11: Correlation heatmap for all possible pairs of tonometric parameters within the PRE group. The colors indicate values of Spearman correlation coefficient (blue for negative correlation, white for no correlation, and red for the positive relationship).

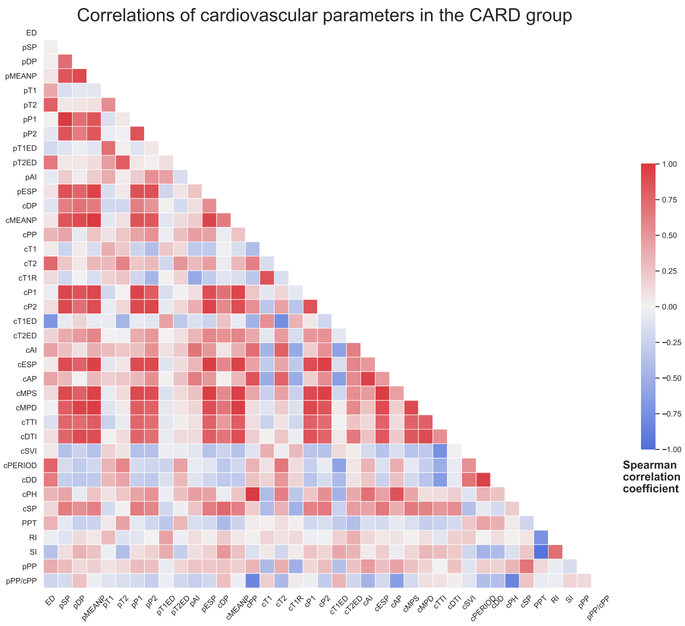

Figure S12: Correlation heatmap for all possible pairs of tonometric parameters within the CARD group. The colors indicate values of Spearman correlation coefficient (blue for negative correlation, white for no correlation, and red for the positive relationship).

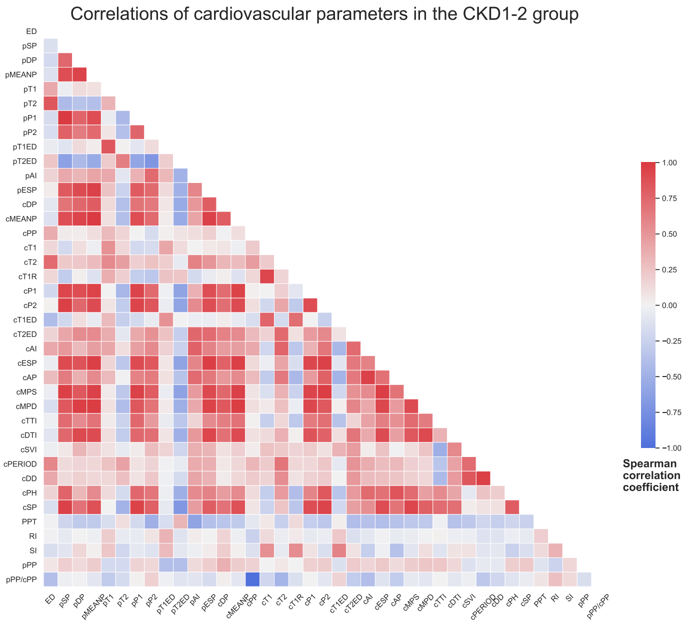

Figure S13: Correlation heatmap for all possible pairs of tonometric parameters within the CKD1-2 group. The colors indicate values of Spearman correlation coefficient (blue for negative correlation, white for no correlation, and red for the positive relationship).

Table S1: Wald test results for the chosen combination of tonometry variables in the multinomial logistic regression model. When z value is away from 0, it is likely that excluding the attached parameter will make the model significantly less precise. As the model itself, the values of z with corresponding p values are calculated for each group (CKD1-2, PD, HD, CARD, PRE) with respect to the Control group.

	**CKD1-2**		**PD**		**HD**		**CARD**		**PRE**	
	z	p	z	p	z	p	z	p	z	p
Intercept	-57.17	$< 10^{-4}$	-2.63	0.0085	11.73	$< 10^{-4}$	-0.42	0.6713	18.11	$< 10^{-4}$
ED	145.35	$< 10^{-4}$	34.42	$< 10^{-4}$	59.05	$< 10^{-4}$	29.52	$< 10^{-4}$	23.78	$< 10^{-4}$
pDP	216.59	$< 10^{-4}$	73.79	$< 10^{-4}$	31.54	$< 10^{-4}$	28.92	$< 10^{-4}$	10.60	$< 10^{-4}$
pT2	-20.27	$< 10^{-4}$	-18.96	$< 10^{-4}$	-14.24	$< 10^{-4}$	-32.84	$< 10^{-4}$	-11.66	$< 10^{-4}$
pT1ED	23.71	$< 10^{-4}$	6.59	$< 10^{-4}$	-0.20	0.8448	33.45	$< 10^{-4}$	5.89	$< 10^{-4}$
pAI	-15.31	$< 10^{-4}$	45.23	$< 10^{-4}$	22.13	$< 10^{-4}$	27.96	$< 10^{-4}$	-11.01	$< 10^{-4}$
cDP	100.19	$< 10^{-4}$	-26.29	$< 10^{-4}$	-13.97	$< 10^{-4}$	-42.38	$< 10^{-4}$	-19.27	$< 10^{-4}$
cPP	1146.39	$< 10^{-4}$	56.20	$< 10^{-4}$	152.62	$< 10^{-4}$	71.98	$< 10^{-4}$	43.95	$< 10^{-4}$
cT1	-145.67	$< 10^{-4}$	-13.59	$< 10^{-4}$	-72.45	$< 10^{-4}$	-93.06	$< 10^{-4}$	14.48	$< 10^{-4}$
cT1R	-306.48	$< 10^{-4}$	-37.26	$< 10^{-4}$	-9.69	$< 10^{-4}$	5.89	$< 10^{-4}$	-12.02	$< 10^{-4}$
cT1ED	289.96	$< 10^{-4}$	-7.04	$< 10^{-4}$	134.57	$< 10^{-4}$	35.37	$< 10^{-4}$	-15.77	$< 10^{-4}$
cAI	-40.95	$< 10^{-4}$	-97.73	$< 10^{-4}$	35.23	$< 10^{-4}$	-152.82	$< 10^{-4}$	38.95	$< 10^{-4}$
cESP	20.71	$< 10^{-4}$	-52.41	$< 10^{-4}$	-78.75	$< 10^{-4}$	-25.10	$< 10^{-4}$	26.43	$< 10^{-4}$
cAP	-244.03	$< 10^{-4}$	-42.94	$< 10^{-4}$	-225.66	$< 10^{-4}$	35.41	$< 10^{-4}$	-54.74	$< 10^{-4}$
cMPD	-66.67	$< 10^{-4}$	18.51	$< 10^{-4}$	-2.13	0.0328	-16.28	$< 10^{-4}$	-23.70	$< 10^{-4}$
cTTI	8.05	$< 10^{-4}$	61.62	$< 10^{-4}$	70.01	$< 10^{-4}$	109.12	$< 10^{-4}$	-67.53	$< 10^{-4}$
cPERIOD	-33.71	$< 10^{-4}$	9.92	$< 10^{-4}$	7.10	$< 10^{-4}$	73.38	$< 10^{-4}$	-34.85	$< 10^{-4}$
cPH	415.27	$< 10^{-4}$	-35.60	$< 10^{-4}$	36.44	$< 10^{-4}$	-177.23	$< 10^{-4}$	54.05	$< 10^{-4}$
cSP	-387.04	$< 10^{-4}$	26.30	$< 10^{-4}$	-4.35	$< 10^{-4}$	135.55	$< 10^{-4}$	25.59	$< 10^{-4}$
PPT	-15.99	$< 10^{-4}$	-1.88	0.0606	-5.30	$< 10^{-4}$	-2.66	0.0078	4.74	$< 10^{-4}$
RI	-8.80	$< 10^{-4}$	16.99	$< 10^{-4}$	21.26	$< 10^{-4}$	7.45	$< 10^{-4}$	-0.14	0.8896
SI	2.15	0.0315	-6.10	$< 10^{-4}$	-11.31	$< 10^{-4}$	4.09	$< 10^{-4}$	7.88	$< 10^{-4}$
pPP	-196.28	$< 10^{-4}$	4.44	$< 10^{-4}$	-17.60	$< 10^{-4}$	-2.88	0.0039	-6.81	$< 10^{-4}$
pPP/cPP	430.10	$< 10^{-4}$	-28.84	$< 10^{-4}$	27.85	$< 10^{-4}$	-59.88	$< 10^{-4}$	-3.49	0.0005

Table S2: Comparison of different available kernels for KFDA algorithm. Accuracy has been calculated by taking an average of 10 classification trials on test data that was randomly selected 20% of patients and hidden during model training. Mean variance within groups has been calculated on normalized model transformed data as an average variance for all groups. The mean Euclidean distance is an average of distances between group midpoints in normalized model space. It is believed that good model shall have high accuracy, low variance within groups and long average distance between midpoints.

**Kernel**	**linear**	**polynomial**	**gaussian**	**sigmoid**	**laplacian**
Accuracy	0.6847 ± 0.0867	0.721 ± 0.0503	0.6979 ± 0.06	0.6642 ± 0.0647	0.8 ± 0.083
Mean variance within the groups	0.1170 ± 0.0179	0.0266 ± 0.0273	0.0259 ± 0.0281	0.0386 ± 0.0309	0.0224 ± 0.0265
Mean Euclidean distance between group centers	0.4030 ± 0.0659	1.0183 ± 0.1972	1.0648 ± 0.2191	1.0014 ± 0.1675	1.0596 ± 0.1652
